# Optimization of Preemptive Therapy for Cytomegalovirus Infections With Valganciclovir Based on Therapeutic Drug Monitoring: Protocol for a Phase II, Single-Center, Single-Arm Trial

**DOI:** 10.2196/72549

**Published:** 2025-06-24

**Authors:** Naoki Tamura, Kotaro Itohara, Yo Ueda, Yumi Kitahiro, Kazuhiro Yamamoto, Tomohiro Omura, Toshiyasu Sakane, Jun Saegusa, Ikuko Yano

**Affiliations:** 1 Department of Pharmacy Kobe University Hospital Kobe Japan; 2 Department of Pharmaceutical Technology Kobe Pharmaceutical University Kobe Japan; 3 Department of Rheumatology and Clinical Immunology Kobe University Graduate School of Medicine Kobe Japan; 4 Department of Integrated Clinical and Basic Pharmaceutical Sciences Faculty of Medicine, Dentistry and Pharmaceutical Sciences Okayama University Okayama Japan

**Keywords:** valganciclovir, ganciclovir, cytomegalovirus, therapeutic drug monitoring, preemptive therapy, dried blood spots

## Abstract

**Background:**

Valganciclovir (VGCV) is the first-line drug for preemptive therapy of cytomegalovirus (CMV) infections. However, even when administered at the dose specified in the package insert, there is significant interindividual variability in the plasma concentrations of ganciclovir (GCV). In addition, correlations have been reported between the area under the concentration–time curve and therapeutic efficacy or adverse events. Therefore, therapeutic drug monitoring (TDM) can be used to improve the efficacy and safety of preemptive VGCV therapy.

**Objective:**

This study aims to evaluate whether the dosage adjustment of VGCV based on TDM in patients undergoing preemptive therapy for CMV infections is associated with the successful completion rate of treatment without severe hematological adverse effects.

**Methods:**

This phase II, single-center, single-arm trial aims to enroll 40 patients admitted at the Department of Rheumatology and Clinical Immunology, Kobe University Hospital, who will receive oral VGCV as preemptive therapy for CMV infections. Participants will begin treatment with VGCV at the dose recommended in the package insert, with subsequent dose adjustments based on weekly TDM results. The primary end point will be the proportion of patients who achieve CMV antigenemia negativity within 3 weeks without severe hematological adverse events. The secondary end points will include weekly changes in CMV antigen levels, total VGCV dose, and duration of preemptive therapy. For safety evaluation, the occurrence, type, and severity of VGCV-related adverse events will be analyzed. Additionally, this study will explore the correlations between the efficacy and safety of preemptive therapy and the pharmacokinetic parameters of GCV, CMV-polymerase chain reaction values, and nudix hydrolase 15 (*NUDT15*) genetic polymorphisms. The correlation between GCV plasma concentrations obtained from regular venous blood and blood concentrations will be examined using dried blood spots.

**Results:**

This study began with patient recruitment in September 2024, with 5 participants enrolled as of June 16, 2025. The target enrollment is 40 participants, and the anticipated study completion is set for July 2027.

**Conclusions:**

This is the first study to investigate the impact of TDM intervention in patients receiving VGCV as preemptive therapy. The findings are postulated to provide valuable evidence regarding the utility of TDM in patients receiving VGCV as preemptive therapy.

**Trial Registration:**

Japan Registry of Clinical Trials jRCTs051240080; https://jrct.mhlw.go.jp/latest-detail/jRCTs051240080

**International Registered Report Identifier (IRRID):**

DERR1-10.2196/72549

## Introduction

### Background

Cytomegalovirus (CMV) establishes a latent infection after the resolution of acute infection. Its reactivation can occur in patients undergoing immunosuppressive therapy, leading to organ-specific infections such as febrile illness, pneumonia, and retinitis [1]. For patients at high risk of CMV infection, their viral load is regularly monitored. Preemptive therapy is initiated once the viral load becomes detectable, even in the absence of symptoms. Preemptive therapy is effective in preventing the onset of CMV infection and is recommended for patients undergoing hematopoietic stem cell transplant [2-4].

Valganciclovir (VGCV) is the first-line treatment not only for the prevention and treatment of CMV infections but also for preemptive therapy. It is rapidly metabolized to its active metabolite ganciclovir (GCV). Because GCV is primarily excreted by the kidneys, the dose of VGCV is adjusted based on renal function [5]. In patients who have undergone solid organ transplant, a correlation has been reported between the therapeutic effect of GCV on CMV viremia and the area under the concentration–time curve (AUC) [6]. Therefore, sufficient AUC exposure is necessary for adequate treatment. Additionally, the relationship between AUC and adverse effects has been reported [6-9]. These reports suggest that the effective range of GCV is between 40 and 60 μg·h/mL of AUC. However, a significant interindividual variability has been observed in the plasma concentrations of GCV, even when administering the dose specified in the package insert [6,10]. To this end, dosage adjustments based solely on the package insert are insufficient, and the usefulness of therapeutic drug monitoring (TDM) for GCV has been reported [7]. However, the utility of routine GCV concentration measurements and TDM remains controversial and is not currently being conducted [11].

Recently, dried blood spot (DBS) sampling, which involves collecting a small amount of blood from the fingertip onto a filter paper, has been increasingly performed in clinical practice as a less invasive method to facilitate TDM [12,13]. In addition, nudix hydrolase 15 *(NUDT15*) polymorphisms were reported to influence the antiviral efficacy and cytotoxicity of GCV [14]. However, the clinical utility of DBS and measuring *NUDT15* polymorphisms for VGCV treatment has not yet been established.

### Study Objectives

The primary objective of this study is to evaluate whether the dosage adjustment of VGCV based on TDM in patients undergoing preemptive therapy for CMV is associated with the completion ratio of treatment without adverse hematological effects. Additionally, this study aims to investigate whether dosage adjustments based on TDM can reduce the time required for CMV pp65 negativity or the incidence rate of adverse effects. Factors related to the pharmacokinetics of GCV and the efficacy and safety of preemptive therapy with VGCV will also be investigated. Further, this study will explore the relationship between GCV plasma concentrations obtained from venous blood and DBS whole blood concentrations at each measurement point and the relationship between *NUDT15* genetic polymorphisms and adverse events. Moreover, an exploratory analysis will be conducted to determine the relationship between CMV antigen quantification using CMV pp65 and CMV viral load measured by quantitative CMV DNA polymerase chain reaction (PCR).

## Methods

### Study Design and Study Location

**Figure 1:**
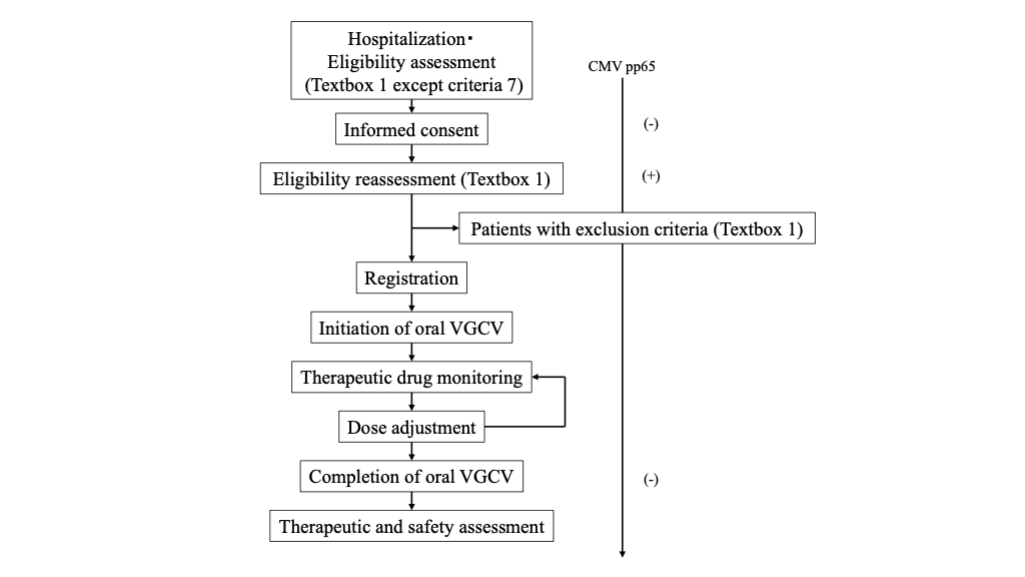
Flowchart of the study design. CMV: cytomegalovirus; VGCV: valganciclovir.

This is a phase II, single-center, single-arm interventional trial. A flowchart of the study design is shown in Figure 1. This study is being conducted at Kobe University Hospital.

### Inclusion and Exclusion Criteria

The inclusion and exclusion criteria are presented in Textbox 1. Patients who meet inclusion criteria 1-6 will be asked by their attending physician to provide informed consent for trial participation. When the weekly measured CMV pp65 levels meet inclusion criterion 7, patients who provide written informed consent will be enrolled in this trial after confirming that they have not withdrawn their consent and still meet all the criteria.

**Study inclusion and exclusion criteria.**

**Inclusion criteria**

1. Inpatients at the Department of Rheumatology and Clinical Immunology
2. Treated with prednisolone more than or equal to 0.5 mg/kg with/without immunosuppressive agents as initial therapy
3. Aged 18 years or older at the time of consent
4. Written informed consent has been obtained regarding their voluntary participation in this clinical study
5. No symptoms due to cytomegalovirus (CMV) infection (eg, fever, cough, dyspnea, abdominal pain, diarrhea, bloody stools, dysopsia)
6. Not received valganciclovir (VGCV) therapy for at least 2 weeks before starting this clinical study
7. Test positive for CMV pp65 antigen and meet one of the following conditions: (1) CMV pp65 antigen is more than or equal to 10 per 2 slides or (2) CMV pp65 antigen is more than or equal to 1 per 2 slides and less than or equal to 9 per 2 slides, and the retest result after 2 to 8 days is that CMV pp65 antigen is more than or equal to 5 per 2 slides and higher than the initial value.

**Exclusion criteria**

1. Patients undergoing dialysis
2. Significant bone marrow suppression such as neutrophil count less than 500/mm^3^ or platelet count less than 25,000/mm^3^
3. Pregnancy or possible pregnancy
4. Known allergies or drug sensitivities to VGCV, ganciclovir, acyclovir, or valacyclovir
5. Hemoglobin levels less than 8.0 g/dL
6. Taking probenecid
7. The investigator deems inappropriate

### Intervention

The study schedule is presented in Table 1. Patients will receive VGCV orally at the dosage specified in the package insert, based on creatinine clearance (Ccr) calculated using the Cockcroft-Gault equation [15]. On day 3 following the initiation of VGCV therapy, venous blood samples will be collected before and 2 hours after the morning dose of VGCV. The plasma concentrations will be measured using liquid chromatography-tandem mass spectrometry [16].

**Table 1:** Summary of the study schedule.

		Hospitalization	Preobservation period	Treatment period									Closeout		Postobservation period
				Week 1					Weeks 2-3						
				Day 1	Day 3	Day 4	Day 5	Day 1		Day 2	Day 3				
**Enrollment**															
	Eligibility screening	✓	✓	✓											
	Informed consent	✓													
	Registration			✓											
**Intervention**															
	Blood sampling for TDM^a^				✓			✓							
	TDM					✓				✓					
	Dose adjustment						✓				✓				
	Blood sampling for *NUDT15*^b^ genotyping											✓			
**Assessments**															
	Patient’s background^c^		✓												
	CMV^d^ pp65/CMV-PCR^e^		✓					✓				✓		✓	
	Laboratory data^f^		✓	✓	✓	✓		✓				✓		✓	
	Adverse events			✓	✓	✓	✓	✓		✓	✓	✓		✓	
	Concomitant medication		✓	✓	✓	✓	✓	✓		✓	✓	✓		✓	

^a^TDM: therapeutic drug monitoring.

^b^*NUDT15*: nudix hydrolase 15.

^c^Including sex, age, height, weight, medical history, complications, and concomitant medications.

^d^CMV: cytomegalovirus.

^e^PCR: polymerase chain reaction.

^f^Including levels of white blood cell, neutrophil, hemoglobin, platelet, hematocrit, aspartate aminotransferase, alanine aminotransferase, albumin, blood urea nitrogen, serum creatinine, estimated glomerular filtration rate, sodium, potassium, and chloride.

### Estimated Apparent Clearance

Individual estimated apparent clearance (CL/F) will be estimated using the post hoc Bayesian analysis based on a previously reported population pharmacokinetic model [17] as follows:

CL/F=7.09*(1+Ccr/68.3) (L/h)

Vc/F=10.8 (L)

Q/F=3.96 (L/h)

Vp/F=174 (L)

Ka=0.23 (/h)

T_lag_=0.93 (h)

where Vc/F, Q/F, Vp/F, Ka, and T_lag_ are the apparent volumes of distribution in the central compartment, apparent intercompartmental clearance, apparent volume of distribution in the peripheral compartment, absorption rate constant, and lag time, respectively. Additionally, interindividual variabilities of CL/F, Vc/F, Q/F, and Vp/F are 27.2, 153, 63.1, and 107%, respectively. Interindividual variabilities of Ka and T_lag_ are not included. The proportional residual variability is 42.9%. The individual AUC will be calculated using the following formula: AUC = daily dose of VGCV × (GCV molecular weight of 255.2/ VGCV molecular weight of 354.4) / estimated individual CL/F. Subsequently, the dosage will be adjusted to achieve the target AUC (40-60 μg·h/mL) by using the dose adjustment shown in Table 2. Patients will be informed of the possibility of dosing higher or lower than the package insert recommendation during the consent process. Thereafter, weekly plasma concentration measurements and dosage adjustments will be performed using the same procedure as described above, with a treatment period of up to 3 weeks. Post hoc Bayesian estimation will be performed using NONMEM version 7.5.1 (ICON).

**Table 2:** Adjusted dosage of valganciclovir based on the estimated apparent clearance.

CL/F^a^ (L/h)	Adjusted dose
2.7^b^ < CL/F ≤ 4.1	450 mg once daily every other day
4.1 < CL/F ≤ 5.4	450 mg once daily for 2 days and 1 day off
5.4 < CL/F ≤ 8.1	450 mg once daily
8.1 < CL/F ≤ 12.2	450 mg twice daily and 450 mg once daily alternately
12.2 < CL/F ≤ 16.2	450 mg twice daily
16.2 < CL/F ≤ 24.3	900 mg in the morning and 450 mg in the evening
24.3 < CL/F ≤ 32.4	900 mg twice daily
32.4 < CL/F ≤ 40.5	1350 mg in the morning and 900 mg in the evening
40.5 < CL/F ≤ 48.6	1350 mg twice daily

^a^CL/F: estimated apparent clearance.

^b^For patients with CL/F less than or equal to 2.7 or greater than 48.6, consider dosage individually.

Protocol treatment will continue for at least 2 weeks up to a maximum of 3 weeks from the day of VGCV initiation until CMV pp65 is confirmed to be negative. If CMV pp65 is not confirmed to be negative at 3 weeks after the initiation of VGCV, posttreatment will be performed at the discretion of the physician. Additionally, DBS sampling will be conducted 2 hours after VGCV morning dose and before each meal on day 3 after VGCV initiation to investigate the correlation between DBS-derived whole blood concentrations and plasma concentrations. Finger-prick blood will be collected onto filter papers (Whatman 903 Protein saver card), and blood collection will be conducted to ensure a sampling diameter of at least 6 mm. Genetic polymorphisms of *NUDT15* will also be analyzed using blood samples collected in this study. DNA will be extracted from blood samples, and *NUDT15* polymorphisms in c.415C>T; rs116855232 (p.R139C) and c.416G>A; rs147390019 (p.R139H) will be genotyped using a TaqMan genotyping assay (ID: C_154823200_10 and C_162697298_10). The postobservation period will be 2 weeks after the completion of VGCV administration to assess adverse events and relapse of CMV infection.

### Sample Size Calculation

A preliminary survey was conducted among 20 patients admitted to the Department of Rheumatology and Clinical Immunology at our hospital between January 2020 and August 2022. The proportion of patients who were unable to complete treatment with hematological toxicities was 65% (13/20). None of the patients failed to complete the treatment because of nonhematological adverse events. The proportion of patients who could not complete treatment for reasons other than adverse events was 5% (1/20). Except for the above cases, none of the other patients failed to become CMV pp65–negative within 3 weeks. Approximately 30% of the patients achieved CMV pp65 negativity within 3 weeks without severe hematologic adverse events. The incidence of hematologic toxicities when GCV AUC was maintained at 40-60 μg·h/mL by TDM was estimated to be 36.5%, based on a previous report [9]. Assuming that the rate of treatment incompletion due to reasons other than adverse events or the rate of failure to achieve CMV pp65 negativity within 3 weeks remained at 5%, the estimated rate of patients who would fail to complete treatment owing to adverse events or fail to achieve CMV pp65 negativity within 3 weeks of TDM would be 41.5%. Therefore, the proportion of patients expected to achieve CMV pp65 negativity within 3 weeks without adverse events under TDM was assumed to be 58.5%. Under conditions of a 2-sided significance level of 5% and a power of 80%, the required sample size to detect an increase in the proportion of patients completing treatment without adverse events was determined to be 31. Considering potential dropouts, the sample size was set at 40.

### Ethical Considerations

This study was approved by the Clinical Research Ethics Committee of Kobe University (C230016) on June 28, 2024. This trial was registered in Japan Registry of Clinical Trials (jRCTs051240080) on June 28, 2024. All the participants will sign an informed consent form after receiving a detailed explanation of the study from the researchers. All participant data will be deidentified prior to analysis. Participants will not receive any financial compensation for their participation. This study will adhere to the protocols and principles outlined in the Declaration of Helsinki. Any modifications to the protocol will require approval from the ethics committee before implementation. To address potential claims resulting from health issues related to the study, the principal investigator has secured clinical research insurance covering compensation for death, serious disability, medical expenses, and medical benefits.

### Outcomes

#### Primary End Point

The primary end point of this study is to determine whether preemptive therapy with VGCV for CMV reactivation could achieve CMV antigenemia negativity within 3 weeks without severe adverse events. Severe adverse events are defined as leukopenia, neutropenia, anemia, and thrombocytopenia classified as grade 3 or higher according to the Common Terminology Criteria for Adverse Events (CTCAE) version 5.0.

#### Secondary End Points

Secondary end points include the change in CMV antigen levels at weekly intervals after VGCV initiation, the total dose of VGCV administered, and the duration of preemptive therapy. Additionally, this study will examine the occurrence, type, and severity of adverse events caused by VGCV for a safety assessment. Furthermore, data that would be relevant to the efficacy or safety of preemptive therapy (estimated AUC at each time point of TDM measurement, Ccr at each time point of TDM measurement, CMV pp65 negativity within 3 weeks of VGCV administration, GCV plasma concentration at each measurement point, total GCV exposure, VGCV dose based on renal function at registration, and CMV antigen repositivity within 2 weeks of completion of treatment), pharmacokinetic parameters of GCV, blood concentration of GCV obtained from DBS, genetic polymorphisms of *NUDT15*, and CMV-PCR value will be collected.

### Statistical Analysis

All analyses will be conducted using R software (version 4.5.1; R Foundation for Statistical Computing).

#### Primary Analysis

A binomial test will be performed for the primary end point. The null hypothesis will be defined as 30% of patients having completed preemptive therapy without severe adverse events. The *P* value will be used as the reference.

#### Secondary Analysis

The number of CMV pp65–positive cells will be examined before and weekly after the initiation of preemptive therapy, and the absolute and relative reductions in the number of these cells compared to baseline will be calculated. Correlation analyses between the absolute or relative reduction in the number of CMV pp65-positive cells and the estimated AUC will also be determined. Further, the median (or mean) total dose of VGCV and the duration of administration will be calculated. The types, severity, and incidence rate of adverse events during the study period will be summarized and stratified by renal function (Ccr≤30, 30<Ccr ≤60, 60<Ccr≤80, Ccr>80 mL/min) or estimated AUC (AUC<40, 40≤AUC≤60, AUC>60 μg·h/mL). Group comparisons will be conducted using Fisher exact test or other appropriate statistical methods.

#### Exploratory Analysis

The median (or mean) of the estimated AUC and dose-adjusted estimated AUC will be calculated for each renal function at the start of treatment (Ccr≤30, 30<Ccr≤60, 60<Ccr≤80, Ccr>80 mL/min). Kruskal-Wallis and Dunn multiple comparisons tests will be performed for each group. A correlation analysis will be conducted between Ccr and the recommended probable dose by using Bayesian estimation. Single and multiple regression analyses with the dose-adjusted estimated AUC as the objective variable and clinical information as the explanatory variables will be performed to explore the factors affecting the dose-adjusted estimated AUC. The proportion of CMV antigen-positive cells that disappear in less than 3 weeks of treatment will be calculated, and a binomial test will be performed against the proportions in the preliminary study. The patients will be grouped according to the presence or absence of disease or CMV infection recurrence, and the median (or mean) of the estimated trough blood concentration, estimated AUC, and total exposure will be calculated in each group. If necessary, the Mann-Whitney *U* test will be performed. The actual total dose of VGCV during the preemptive therapy and the total dose assuming that VGCV is administered at the dose stated in the package insert will be calculated, and a Wilcoxon signed-rank test will be performed. Patients will be grouped according to *NUDT15* genotype (wild-type, homozygous variant or heterozygous variant), and the number and proportion of adverse events in each group will be calculated. Fisher exact test will be performed. Additionally, the median (or mean) estimated trough plasma concentration, estimated AUC, and total exposure will be calculated for each group, and a Mann-Whitney *U* test will be conducted. If both CMV pp65 antigen and CMV-PCR are positive, the correlation will be analyzed using the Spearman rank correlation coefficient. *P* values calculated from the above analyses will be used as references.

Correlation analysis will be performed between plasma obtained by regular venous blood sampling and whole blood concentrations obtained via DBS. A preliminary analysis will be performed in 20 cases, which is half the sample size. If no clear correlation is determined, blood sampling using the DBS method will be discontinued in subsequent studies. Additionally, a simple regression analysis will be conducted with DBS whole blood concentration as the dependent variable and conventional plasma concentration measurements and clinical information as the independent variables.

#### Interim Analysis

After 20 cases, half of the sample size would have been enrolled, and the efficacy and occurrence of serious adverse events will be summarized. Regarding the efficacy, the probability of the effectiveness of TDM when the remaining cases are enrolled will be evaluated using a binomial distribution. Considering this probability and the incidence rate or grades of serious adverse events, we will decide whether the trial should be continued or discontinued based on a comprehensive assessment. To allow for flexibility, no specific statistical threshold is set in this decision-making process.

### Data Collection and Management

After receiving consent from prospective patients, the investigators will assess their eligibility. To this end, the research secretariat will access the Research Electronic Data Capture (an electronic data system for clinical research; REDCap) system to confirm eligibility before inputting essential information for case registration and assigning a case number. The registration process will conclude upon the display of a confirmation of the case registration.

### Data Monitoring

This study will undergo regular monitoring to safeguard human rights and welfare. It will be conducted with strict adherence to the protocol and all relevant regulatory requirements to ensure safety. The principal investigator has designated the individuals responsible for overseeing the procedures outlined for study monitoring. To ensure quality control, the monitor will assess adherence to the protocol and outline the procedures during the study.

## Results

Recruitment for this study began in September 2024, and as of June 16, 2025, 5 participants have been enrolled. Study completion is anticipated by July 2027. The study protocol and statistical analysis plan will be made available to the Japan Registry of Clinical Trials. The findings of this study will be presented at medical conferences and published in scientific papers. After publication, the corresponding author will make the collected data accessible in a nonpersonally identifiable form upon reasonable request from other researchers within a prescribed period.

## Discussion

To the best of our knowledge, this is the first study that will investigate the impact of TDM intervention on improving the completion rate of preemptive therapy in patients receiving VGCV. In this study, both patients and investigators are aware of dose adjustments. However, the efficacy and safety of the VGCV therapy will be evaluated objectively by clinical laboratory values and CTCAE version 5.0. Therefore, observer and confirmation bias will not occur.

The use of VGCV as preemptive therapy is not specified in its package insert. Still, it is common based on previous reports in patients who undergo hematopoietic stem cell transplant [2-4], suggesting its effectiveness in clinical practice in Japan. GCV is known to have large interindividual variability in plasma concentrations after oral administration of VGCV. A previous report indicated that patients with impaired renal function experience higher exposure to GCV, whereas those with better renal function experience lower exposure [7]. High plasma concentrations of GCV are associated with an increased frequency of adverse events, whereas low concentrations would lead to insufficient therapeutic efficacy. Therefore, achieving the target AUC through TDM would increase both safety and efficacy.

In solid organ transplant recipients, the association between GCV AUC and the reduction in the incidence of CMV viremia has been reported (AUC 46.3, SD 15.2 μg·h/mL: 2.9%; AUC 28.0, SD 10.9 μg·h/mL: 10.4%) [10]. Furthermore, higher steady-state AUC levels have been shown to result in lower incidence rates of CMV viremia during and up to 1 month after prophylactic treatment, indicating superior preventative efficacy [10]. In preemptive therapy as well, studies have reported a correlation between GCV AUC and treatment outcomes for CMV viremia, with optimal therapeutic effects observed at an AUC in the range of 40-50 μg·h/mL [7]. Thus, sufficient AUC exposure is necessary to ensure adequate therapeutic intensity. However, an association between GCV AUC and adverse event incidents have been documented. For example, the incidence of hemoglobin reduction was reported to be 51.9% at an AUC >50 μg·h/mL compared to 26.6% at an AUC ≤50 μg·h/mL [7]. Additionally, the incidence of leukopenia and neutropenia increased with increasing AUC levels. For leukopenia of grade 1 or higher, the incidence rate exceeded 50% at an AUC ≥60 μg·h/mL [10,11]. Therefore, from the perspective of efficacy and safety, a target AUC of 40-60 μg·h/mL is often recommended. Based on these findings, the target AUC in this study will be set at 40-60 μg·h/mL. The concomitant immunosuppressants may independently affect the incidence of CMV reactivation and/or hematological toxicity. Therefore, the concomitant medication data will be collected for the entire study period and consider them as adjustment factors in the analysis if necessary.

AUC is considered as an indicator of the efficacy and safety of GCV, and many blood collection points are required to accurately determine it, which results in significant burden for the patient. In this study, Bayesian estimation using a population pharmacokinetic model aimed at achieving target AUCs was considered useful for TDM with GCV. Several population pharmacokinetic models of GCV after VGCV administration have been reported [10,17-22], but none have been developed in Japan by using rich sampling data. Therefore, we decided to use a model developed for the Chinese population because of its racial proximity [17]. The selected report showed that the optimal sampling points were two (0 and 2 hours) or three (0, 2, and 4 hours) after VGCV administration [17]. In this study, the sampling points were set at the trough and 2 hours after VGCV administration to reduce patient burden.

To ensure high predictive accuracy for the AUC by Bayesian estimation, we required multiple blood samples. DBS sampling has the advantage that blood samples can be collected in a nonclinical setting such as at home, and only a small amount of blood is required for measurement. As multiple blood samples are required to calculate AUC, replacing venous blood sampling with DBS could reduce the physical burden on patients. However, the correlation between plasma concentrations measured by venous blood sampling and DBS whole blood concentrations remains unclear and investigating this relationship could contribute to less invasive TDM interventions.

Recently, pharmacogenomics, which investigates how genetic polymorphisms of metabolic enzymes or transporters influence the variability in drug responses, has attracted attention as a useful tool in personalized medicine. *NUDT15* polymorphisms are involved in the metabolism of thiopurine drugs. Genetic testing for codon 139 polymorphisms is useful for predicting acute severe adverse events for patients with refractory inflammatory bowel disease, acute lymphoblastic leukemia, or other conditions treated with thiopurine drugs [23]. Although recent reports suggest that *NUDT15* polymorphisms influence not only the antiviral efficacy but also the cytotoxic effects of GCV and acyclovir in vivo [14], no human studies have yet been conducted. The exploratory research in this study could provide useful information on the utility of *NUDT15* genotyping in the treatment of VGCV.

This study has a limitation. Because TDM of GCV is not commonly performed, this study is conducted at a single center to ensure the accuracy and speed of TDM measurements and to obtain an effect size without confounding. Therefore, the findings of this study may not be generalized. Once the usefulness of GCV TDM for preemptive therapy is confirmed, its application, including the impact of lag-time in GCV measurement and diverse patient characteristics at multiple institutions, needs to be clarified in the phase III trial. Overall, this study provides valuable evidence regarding the utility of TDM in patients receiving VGCV as preemptive therapy.

## Abbreviations

AUC: area under the concentration–time curve
Ccr: creatinine clearance
CL/F: estimated apparent clearance
CMV: cytomegalovirus
CTCAE: Common Terminology Criteria for Adverse Events
DBS: dried blood spot
GCV: ganciclovir
PCR: polymerase chain reaction
REDCap: Research Electronic Data Capture
TDM: therapeutic drug monitoring
VGCV: valganciclovir

## Data Availability

The datasets generated or analyzed in this study are available from the corresponding author upon request after publication.
